# Effects of skim milk and isotonic drink consumption before exercise on fluid homeostasis and time-trial performance in cyclists: a randomized cross-over study

**DOI:** 10.1186/s12970-020-00346-9

**Published:** 2020-03-30

**Authors:** Danniela García-Berger, Karen Mackay, Matias Monsalves-Alvarez, Carlos Jorquera, Rodrigo Ramirez-Campillo, Hermann Zbinden-Foncea, Mauricio Castro-Sepulveda

**Affiliations:** 1 Nutrition and Exercise laboratory, Faculty of Medicine, Universidad Mayor, Santiago, Chile; 2 School of Exercise and Nutrition, Faculty of Health, Queensland University of Technology, Brisbane, Australia; 3 Human Performance Laboratory, Motion Health & Performance Center, Santiago, Chile; 4 Laboratory of Human Performance. Quality of Life and Wellness Research Group. Department of Physical Activity Sciences, Universidad de Los Lagos, Osorno, Chile; 5 Exercise Science Laboratory, School of Kinesiology, Faculty of Medicine, Universidad Finis Terrae, Av. Pedro de Valdivia # 1509, Providencia, Santiago, Chile

**Keywords:** Hydration, Sport drink, Milk, Cyclists, Endurance sport, Urine specific gravity

## Abstract

**Background:**

Hydration status affects endurance performance. Pre-exercise hydration recommendations target the consumption of high carbohydrate and sodium beverages. Milk, due to its carbohydrate and sodium content, may be considered an effective pre-exercise hydration beverage.

**Purpose:**

In a randomized cross-over trial, we compared the effects of an isotonic sport drink (SPD) with skim milk (SM) consumption before a race, on fluid homeostasis and time-trial performance in road cyclists.

**Methods:**

Male road cyclists (*n* = 9; age, 26.8 ± 4.78 years) with 10.8 ± 8.56 years of experience in national competitions, consumed either SPD or SM in doses of 350 mL at 3 h and 350 mL at 1.5 h before a 18.6 km time-trial race. Measurements of body mass, urine specific gravity (USG), urine color and time-trial were compared between drinks (group; g) before and after the race (time; t).

**Results:**

The two-way ANOVA showed no differences between SPD and SM in body mass (t, *p* < 0.0001; g, *p* = 0.89; t × g, *p* = 0.54), USG (t, *p* = 0.01; g, *p* = 0.63; t × g, *p* = 0.29) and urine color (t, *p* = 0.01; g, *p* = 0.54; t × g, *p* = 0.28) before or after race. Furthermore, no differences on water consumption during the race (*p* = 0.55) or time-trial performance (*p* = 0.84) were observed between trials.

**Conclusion:**

Current results may help athletes with different beverages preferences to increase their options of hydration strategies.

## Introduction

The position statement of the American College of Sports Medicine on exercise and fluid replacement stated that a hypohydration greater than 2% of body mass impairs endurance exercise performance [1, 2]. Specifically in cyclists, hypohydration has shown to decrease time-trial performance independent of thirst or when there is unknown hydration state [3–6]. Therefore, hydration strategies during and after competition are fundamental to maintain exercise capacity and will contribute to optimal performance during competition, especially in long lasting endurance events [7]. Nevertheless, research involving these strategies are scarce. The interest of pre-exercise hydration recommendations have shown that the consumption of beverages with high amounts of sodium and different types of carbohydrates can reduce fluid loss and improve fluid balance during exercise [1, 8]. Previous research involving pre-exercise consumption of chicken soup [9] and non-alcoholic beer [10] have shown improved fluid balance during exercise. However, none of these studies considered performance.

The use of chocolate skim milk (CSM) has become an alternative to other commercially distributed isotonic sport drinks (SPD). In a literature review article, Pritchett et al. (2012) concluded that consuming CSM immediately after exercise and at 2 h post-exercise, attenuated indices of muscle damage and favored optimal exercise recovery [11]. Moreover, CSM, when compared to other SPD, provided similar or superior effects on ratings of perceived exertion, serum lactate, and serum creatine kinase after exercise [12]. These results suggest that post-exercise hydration drinks could also be effective when used as pre-exercise hydration beverages [1]. So, the use of CSM, could be considered a potential prior to exercise sport drink. However, CSM has almost twice as many calories as any other SPD, which could lead to gastrointestinal discomfort. Liquid gastric emptying depends on the total caloric content of beverages [13]. Thus, the ingestion of CSM prior to exercise could delay gastric emptying and cause discomfort in athletes. However, the use of non-chocolate skim milk (SM) may be an alternative to CSM. SM has lower calories due to lower carbohydrate content but yet similar nutritional content (other than carbohydrate) compared to CSM. Moreover, it also has similar amount of calories when compared to traditional SPD. Therefore, the aim of this study was to compare the use of a isotonic sport drink (SPD) and skim milk (SM) as a pre-race hydration beverage on fluid homeostasis and time-trial performance in road cyclists.

## Materials and methods

### Participants

Nine male road cyclists (age, 26.8 ± 4.78 years; body mass, 71.9 ± 5.33 kg; height, 175.5 ± 4.07 cm), volunteered for the study. The participants had similar training level (>three training sessions per week) and 10.8 ± 8.56 years of experience in national competitions. All of the cyclists were current competitors (i.e., recently competed or training for an upcoming competition) at the time of testing. Also, all athletes were regular consumers of SM and presented no lactose intolerance. They were carefully informed about all the experimental procedures and risks/benefits associated with their participation in the study. Participants were given a written informed consent and signed the document before any of the experimental testing was performed. This study was conducted in accordance with the Declaration of Helsinki and was approved by the ethics committee of University *** blind for review purposes*** (to be completed after peer review).

### Experimental design

**Fig. 1:**
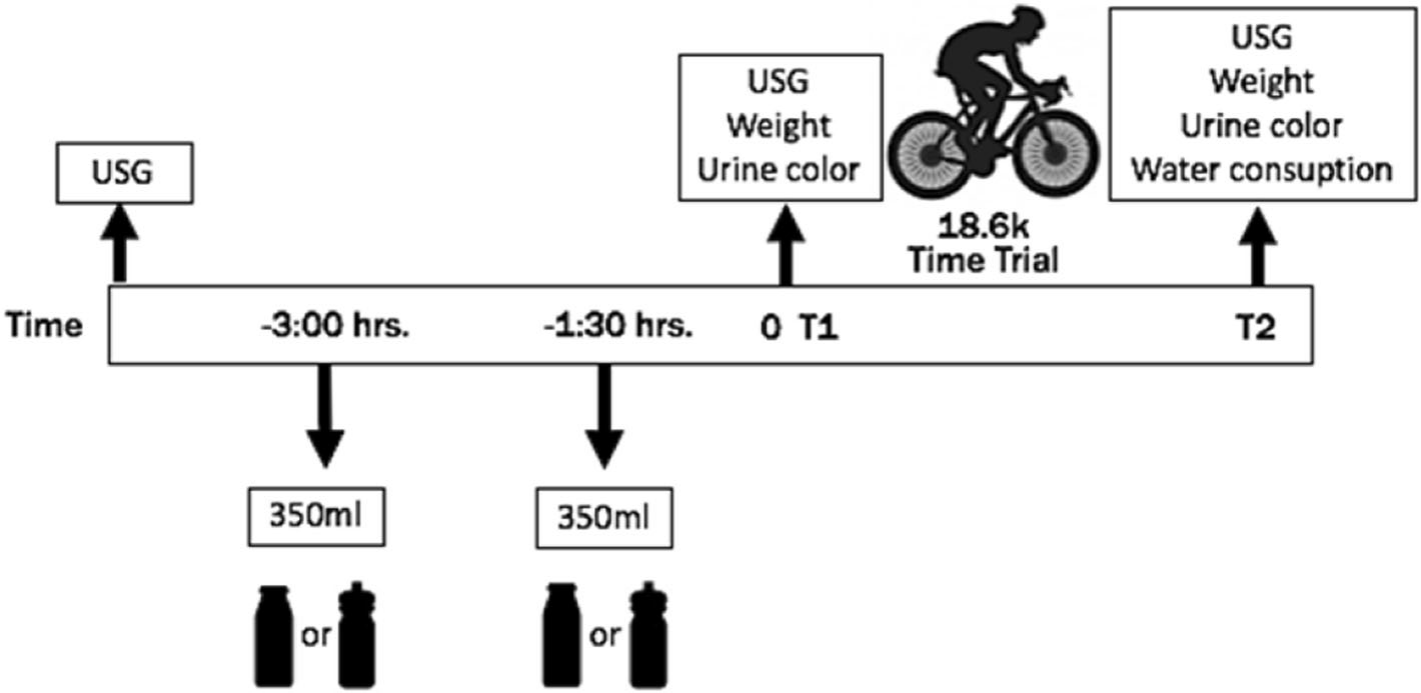
Time chart of the study protocol. USG: Urine specific gravity. T: Time

Each cyclist completed two pre-exercise hydration protocols (5-days of separation between) in a randomized cross-over design, before an 18.6 km time-trial race. The time-trial was conducted on a first category mountain pass which the cyclists were familiar with, and frequently used in cycling competitions. The mountain is located at the Metropolitan Region of Chile (Farellones), with an altitude of ~ 2430 m over sea level and an average gradient of ~ 5%. The time-trial was supervised by the main researcher on an individual basis. To increase the ecological validity, no hydration recommendations were made before the trials. Prior to each trial the athletes ingested two doses of 350 mL (700 mL total), of either SPD (Gatorade®, USA) or SM (Colun®, Chile) (drinks characteristics in Table 1). The doses were given 180 min and 90 min before each race (protocol time-chart in Fig. 1).

**Table 1:** Nutritional characteristics of drinks per 350 mL

Characteristics	Skim milk (SM)	Isotonic sport drink (SPD)
Energy (Kcal)	112	84
Protein (g)	11.5	0
Fat (g)	0.1	0
Carbohydrates (g)	16.4	21
Sodium (mg)	112	185
Potassium (mg)	384	32

### Measurements

Measurements of body mass (SECA, Germany), USG (refractometer; Robinair, USA), and urine color (scale 1 to 8) were assessed after the consumption of 700 mL of the drinks (Time 1) and after the completion of the race (Time 2) (Fig. 1). In addition, baseline USG was also evaluated before drink consumption. For urine collection, a disposable, clean, dry and transparent container was used. Time-trial performance was assessed on each 18.6 km race using a chronometer (Casio, Japan) with precision at normal temperature +/− 99.9%.

To assess liquid intake during the race, 500 mL water bottles (Vital, Chile) were given to each cyclist to be consumed freely during the exercise (ad libitum) at the beginning of the race. Wind, speed, temperature and humidity, was measured using the AccuWeather application. Temperature and humidity were similar (statistical analysis not showed) between SPD (24 °C and 46% of humidity) and SM (21 °C and 48% of humidity).

#### Statistical analysis

Data normality was evaluated with the Shapiro-Wilk test, showing that data was normally distributed. The statistical analysis was performed with GraphPad Prism 6.0 program (Graphpad Software, USA). A paired *t*-test was used to compare water consumption during time-trials and the time needed to complete them. A two-way analysis of variance (2-way ANOVA) was applied to compare body mass, urine color and urine specific gravity before and after race (time) between trials (group) and their interaction (t × g). All data are reported as mean ± standard deviation. *p* < 0.05 was considered statistically significant.

## Results

### Hydration status and fluid homeostasis

**Fig. 2:**
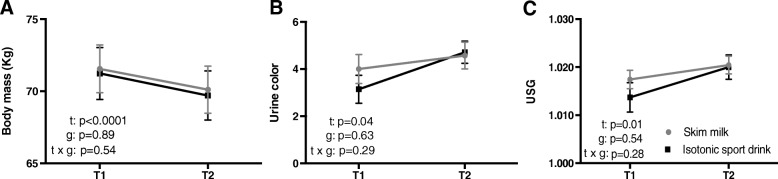
Body mass (**a**), urine color (**b**) and urine specific gravity (USG) (**c**) before (T1) and after (T2) a 18.6 km cycling time-trial race, after ingesting either skim milk (grey line) or isotonic sport drink (black line). Time effects (all *p* < 0.05) were noted for body mass, urine color, and USG, however, no group, and no time × group interactions were noted (see text for further details)

Group baseline USG (before the consumption of drinks) was 1.018 ± 0.006. According to Casa et al. (2000) [7], 11.7% of cyclist were euhydrated (USG < 1.010), 41.2% mildly dehydrated (USG = 1.010—1.020), 41.1% significantly dehydrated (USG = 1.021—1.030), and 5.8% seriously dehydrated (USG > 1.030). Urine color and USG, both hydration status markers, increased after the race (t, *p* = 0.04; t, *p* = 0.01; respectively). However, no differences were found between trials (g, *p* = 0.63; g, *p* = 0.54; respectively), nor in time x trials interaction (t × g, *p* = 0.29; t × g, *p* = 0.28; respectively) (see Fig. 2 b, c). Regarding body mass, both groups showed a reduction of 2.1 ± 0.48% (t, *p* < 0.0001) after the race. However, no differences were found between trials (g, *p* = 0.89), nor in the time x trials interaction (t × g, *p* = 0.54) (see Fig. 2a).

### Water consumption and time-trial performance

**Fig. 3:**
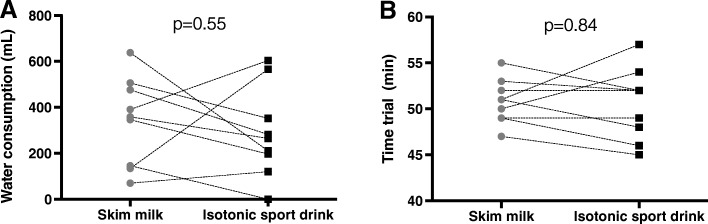
Comparison of water consumption during - and the time needed to complete - a 18.6 km cycling time-trial race, after ingesting either skim milk or isotonic sport drink

No differences were observed on water consumption during the race and between trials (SM, 340 ± 190 mL; SPD, 288 ± 196; *p* = 0.55, see Fig. 3a). Finally, no differences were observed in the 18.6 km time-trial between beverages (SM, 50.6 ± 3.9 min; SPD, 50.8 ± 2.39 min; *p* = 0.84, see Fig. 3b).

## Discussion

The aim of this study was to compare the use of an isotonic sport drink (SPD) and skim milk (SM) as a pre-race hydration beverage on fluid homeostasis and time-trial performance in road cyclists. We observed that SM had similar effects than SPD on fluid homeostasis and performance. Therefore, SM and SPD can be considered as alternatives to hydration drinks prior to a race in road cyclists.

Our results show that 88.2% of the cyclists recruited were dehydrated (USG > 1.010) prior to time-trials. Similar results regarding hydration status on athletes have been reported elsewhere. In a study performed by Castro-Sepúlveda et al. (2015) professional soccer players began training sessions with an average USG of 1.026 ± 0.005 (98% dehydration prevalence) [14]. Furthermore, Díaz-Castro et al. (2018) observed basketball players with an average USG of 1.021 ± 0.006 (98% dehydration prevalence) [15]. Thus, our results of poor hydration prior to competition are in accordance with the literature. It has been suggested that the lack of nutritional knowledge by athletes may be the main cause of a poor hydration status [16–19] prior to competition. Moreover, a recent finding suggests that mild pre-match dehydration (USG = 1.010—1.020) increases the physiological stress during exercise [20]. Therefore, given the high prevalence of pre-exercise dehydration among athletes, pre-hydration strategies and nutritional education among athletes becomes essential.

The literature recommends different hydration protocols prior to exercise [7, 21]. In this study, a total volume intake of 700 ml was provided, divided into two doses of 350 ml, at 3 and 1.5 h prior to the time-trial. This volume was well accepted by the athletes, since no participant declared gastrointestinal discomfort. The literature explains, that most of the gastrointestinal discomfort which occurs during exercise is due to a reduction in gastric emptying speed, malabsorption of water and nutrients, delayed transit time or a reduction in flow splanchnic blood [22]. Most importantly, gastrointestinal discomfort during exercise has shown to impair performance in endurance athletes [22]. Therefore, the prevalence of gastrointestinal discomfort and symptoms during training and competition becomes relevant. It has been suggested that high caloric content in beverages affects gastric emptying. A study by Okabe et al. (2015) compared five types of beverages with different caloric contents (including milk, orange juice and water) and observed no differences in the time of liquid gastric emptying. Therefore, liquid gastric emptying can depend of more nutrients rather than only the total caloric content of the drinks [13]. Our participants reported no gastrointestinal discomfort with the use of SM or SPD. These results support the use of SM as a pre hydration drink, free of any gastrointestinal risks or symptoms.

In the present study, no time differences were found between SM and SPD in the completion of an 18.6 km cycling time-trial. Similar results where showed by Pritchett et al. (2009), where chocolate skim milk and SPD were ingested prior to a time to exhaustion trial at 85% of participant’s maximal oxygen uptake [23]. However, there could be an additional benefit to the use of SM (whether chocolate or not) relative to SPD. The use of SM could contribute in reducing skeletal muscle damage caused by exercise, presumably due to the amino acid content in SM [11]. Further research in this topic is warranted.

In conclusion, our results show that the use of SM as a pre-hydration beverage observed no differences on fluid homeostasis or performance when compared to SPD in trained cyclists. Also, the use of SM presented good gastrointestinal tolerance within the athletes. Therefore, the use of SM could be considered another effective hydration strategy for athletes with different beverages preferences.

## Data Availability

The datasets used and/or analyzed during the current study are available from the corresponding author on reasonable request.

## Abbreviations

CSM: Chocolate skim milk
SM: Skim milk
SPD: Isotonic sport drink
USG: Urine specific gravity
